# Up-Regulation of MicroRNA-21 Correlates with Lower Kidney Cancer Survival

**DOI:** 10.1371/journal.pone.0031060

**Published:** 2012-02-08

**Authors:** Mohd Saif Zaman, Varahram Shahryari, Guoren Deng, Sobha Thamminana, Sharonjot Saini, Shahana Majid, Inik Chang, Hiroshi Hirata, Koji Ueno, Soichiro Yamamura, Kamaldeep Singh, Yuichiro Tanaka, Z. Laura Tabatabai, Rajvir Dahiya

**Affiliations:** Department of Urology, San Francisco Veterans Affairs Medical Center and University of California San Francisco, San Francisco, California, United States of America; The University of Texas M.D Anderson Cancer Center, United States of America

## Abstract

**Background:**

MicroRNA-21 is up-regulated in a variety of cancers like, breast, colorectal, lung, head and neck etc. However, the regulation of miR-21 in renal cell carcinoma (RCC) has not yet been studied systematically.

**Methods and Results:**

We measured miR-21 levels in 54 pairs of kidney cancers and their normal matched tissues by real-time PCR. The expression level of miR-21 was correlated with 5 year survival and the pathological stage. Functional studies were done after inhibiting miR-21 in RCC cell lines. We studied *in vitro* and *in vivo* effects of the chemo preventive agent genistein on miR-21 expression. In 48 cases (90%), miR-21 was increased. All patients with low miR-21 expression survived 5 years, while with high miR-21 expression, only 50% survived. Higher expression of miR-21 is associated with an increase in the stage of renal cancer. Functional studies after inhibiting miRNA-21 in RCC cell lines show cell cycle arrest, induction of apoptosis and reduced invasive and migratory capabilities. Western blot analysis showed an increase in the expression of p21 and p38 MAP kinase genes and a reduction in cyclin E2. Genistein inhibited the expression of miR-21 in A-498 cells and in the tumors formed after injecting genistein treated A-498 cells in nude mice besides inhibiting tumor formation.

**Conclusions:**

The current study shows a clear correlation between miR-21 expression and clinical characteristics of renal cancer. Thus we believe that miR-21 can be used as a tumor marker and its inhibition may prove to be useful in controlling cancers with up-regulated miR-21.

## Introduction

MicroRNAs are small non-coding RNAs that play important roles in numerous cellular processes including development, proliferation, and apoptosis [1]. Since miRNAs are involved in critical cellular processes, recent studies have shown that they are also involved in the pathogenesis of various diseases including those of the kidney. Characteristic miRNA signatures for several epithelial cancers, including breast, lung, pancreatic, gastric and renal cancers, have been reported [2], [3], [4], [5]. Histological evidence shows that there are several variants of malignant renal cell carcinoma, *viz.* clear cell, papillary, chromophobe and collecting duct carcinomas [6], [7], [8], [9]. Several studies have shown differential expression of miRNA in these variants of renal cell carcinoma [10], [11], [12]. MicroRNA-21 is up-regulated in a variety of cancers, such as breast, colorectal, lung, head and neck, etc. However, the regulation and functional role of miR-21 in kidney cancer has not yet been systematically studied. In this study we measured miR-21 levels in 54 pairs of human kidney cancers and their normal matched tissues by real-time PCR. The expression level of miR-21 was correlated with 5 year survival and stage of the patients. In order to determine the role of miR-21 in renal cell carcinoma (RCC) functional assays such as cell cycle, apoptosis, invasion, and migration, were done after knocking down miR-21 expression in A-498 cells. Software analysis and PCR array analysis showed an increase in the expression of p21 and p38 MAP kinases after the inhibition of miR-21 and decreased expression of cyclins and cyclin dependent kinases. These results were validated by Western analysis. Furthermore, we also assessed the effect of a dietary chemoprevention agent, genistein (a soy product), on the expression of miR-21 in mice tumors. Genistein reduced the expression of miR-21 in mice injected with genistein treated cells as compared to vehicle controls. This is the first study which shows the correlation of miR-21 expression with clinical parameters and it's functional role in RCC. In conclusion, up-regulated miR-21 in kidney cancer may be a good tumor marker for kidney cancer diagnosis.

## Results

### miR-21 is up regulated in renal carcinoma tissue samples and renal cancer cell lines

To understand the clinical relevance of miR-21 in kidney cancer we measured miR-21 levels in 54 pairs of kidney cancers and their matched normal tissues by real-time PCR. In 48 cases (89%), miR-21 was found to be increased (T/N [Tumor/Normal] was greater than 1.2). Expression of miR-21 was also measured in renal cancer cell lines, A-498, Caki-1, Caki-2 and normal HK-2 cells. miR-21 expression in A-498 cells was about 7.3× that of HK-2 cells while that of Caki-1 was 1.6×, and Caki-2, 3×.

### Correlation of miR-21 expression with stage and survival in renal cancer

**Figure 1:**
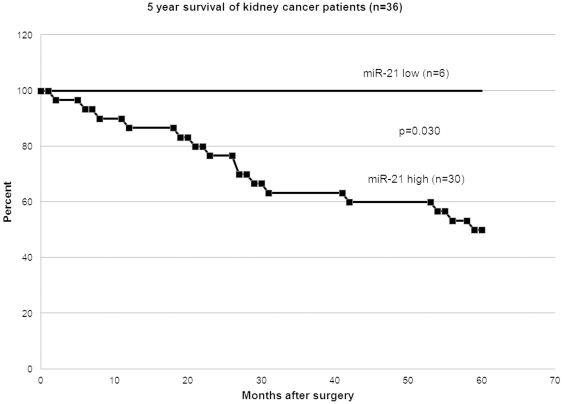
Correlation of miR-21 expression with 5 year survival of renal cell carcinoma patients.

The expression level of miR-21 correlated with 5 year survival and stage of the patients. For patients with low miR-21 expression (Figure S1), all survived 5 years after surgery, while for those with high miR-21 expression (Figure S2), only 50% survived (Figure 1). The survival curve was based on the number of patients (36) for which we had survival information out of a total of 54 patients. In groups of patients having low miR-21 expression (T/N<1.2), high expression (T/N = 1.2–10) and very high expression (T/N>10), the percent of those in early stage (Stage I) decreased by 80%, 51.5% and 37.5%, respectively. This implies that increased expression of miR-21 correlates with an increased stage of renal cancer. This information was based on the number of patients for whom we had the stage information (46 out of 54 patients). No correlation between tumor grade and miRNA-21 levels was observed.

### Expression of miR-21 in different forms of renal cell cancer

The expression of miR-21 was graded as either low (T/N<1.2), high (T/N = 1.2–10) and very high (T/N>10). The different forms of cancers in which the expression of miR-21 was analyzed were clear cell carcinoma, papillary carcinoma and tubular carcinoma. Out of a total of 54 samples, 45 samples were clear cell carcinoma, 8 were papillary and 1 was tubular carcinoma. In both clear cell (71.1%) and papillary carcinoma (75%) the majority of samples had high expression of miR-21 (T/N = 1.2–10). 17.7% of clear cell carcinoma had very high expression. The single tubular renal cell carcinoma sample had very high miR-21 expression.

### Functional role of miR-21 in A-498 cells

To elucidate the functional role of miR-21 in renal cancer we inhibited the expression of miR-21 in A-498 renal cancer cells with a commercially available miR-21 inhibitor. Anti-miR-Negative control #1 was used as the standard negative control for these experiments. The miR-21 level was reduced by more than 99%, as compared to the negative control (Figure S3). In the case of anti-miR-21 transfection into Caki-2 cells the same level of reduction of miR-21 expression was observed (Figure S4).

#### Effect on A-498 cell cycle and apoptosis

**Figure 2:**
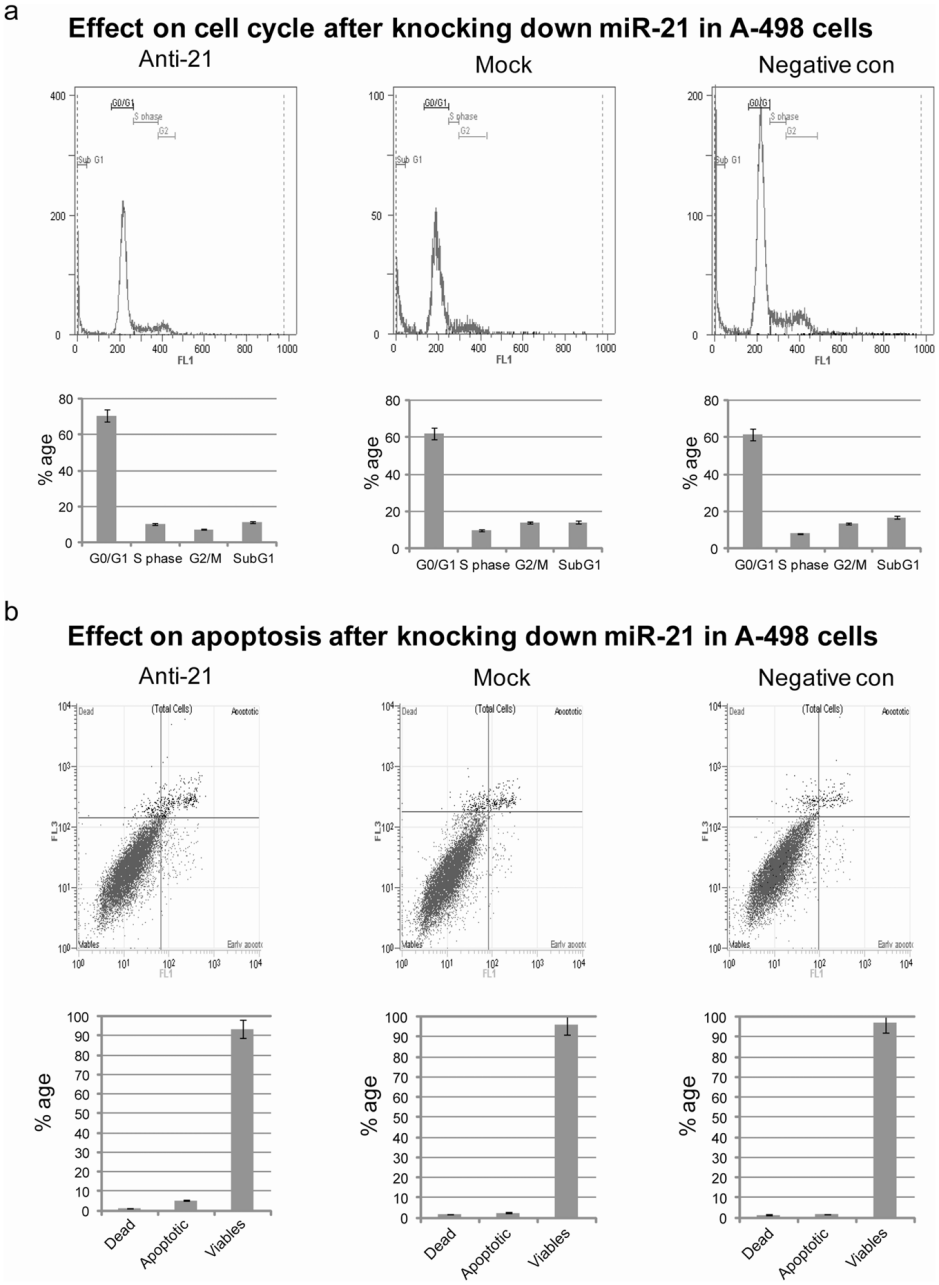
Effect on cell cycle and apoptosis after knocking down miR-21 in A-498 cells. (a) Effect on cell cycle: Flow cytometry analysis of the cell cycle showed a significant increase in G0/G1 phase (9.1%), with a concomitant reduction in the G2/M phase (6.8%), indicating that the forced reduction of miR-21 expression in A-498 cells could inhibit the cell growth and cause G0/G1 arrest (p value 0.010). (b) Effect on apoptosis: Apoptosis assay showed a marginal increase in the number of total apoptotic cells (5.17%) in the miR-21 anti-miRNA inhibitor transfected cells as compared to the negative control (1.79%) and mock (2.49%) (p value 0.017).

The effects of miR-21 inhibition on the cell cycle and apoptosis were analyzed by flow cytometry. The cell cycle showed a significant increase (9.1%) in the G0/G1 phase, with a concomitant reduction (6.8%) in the G2/M phase, indicating that the reduction of miR-21 expression in A-498 cells inhibited the cell growth and caused G0/G1 arrest (Figure 2a). Apoptosis assay using flow cytometry showed a marginal increase in the number of total apoptotic cells (early apoptotic plus apoptotic) (5.17%) in the miR-21 anti-miRNA inhibitor transfected cells as compared to the negative control (1.79%) and mock (2.49%) (Figure 2b). In case of Caki-2 cells inhibition of miR-21 resulted in a significant increase in apoptosis with no change in cell cycle (data not shown).

#### Effect on cell invasion and migration properties

**Figure 3:**
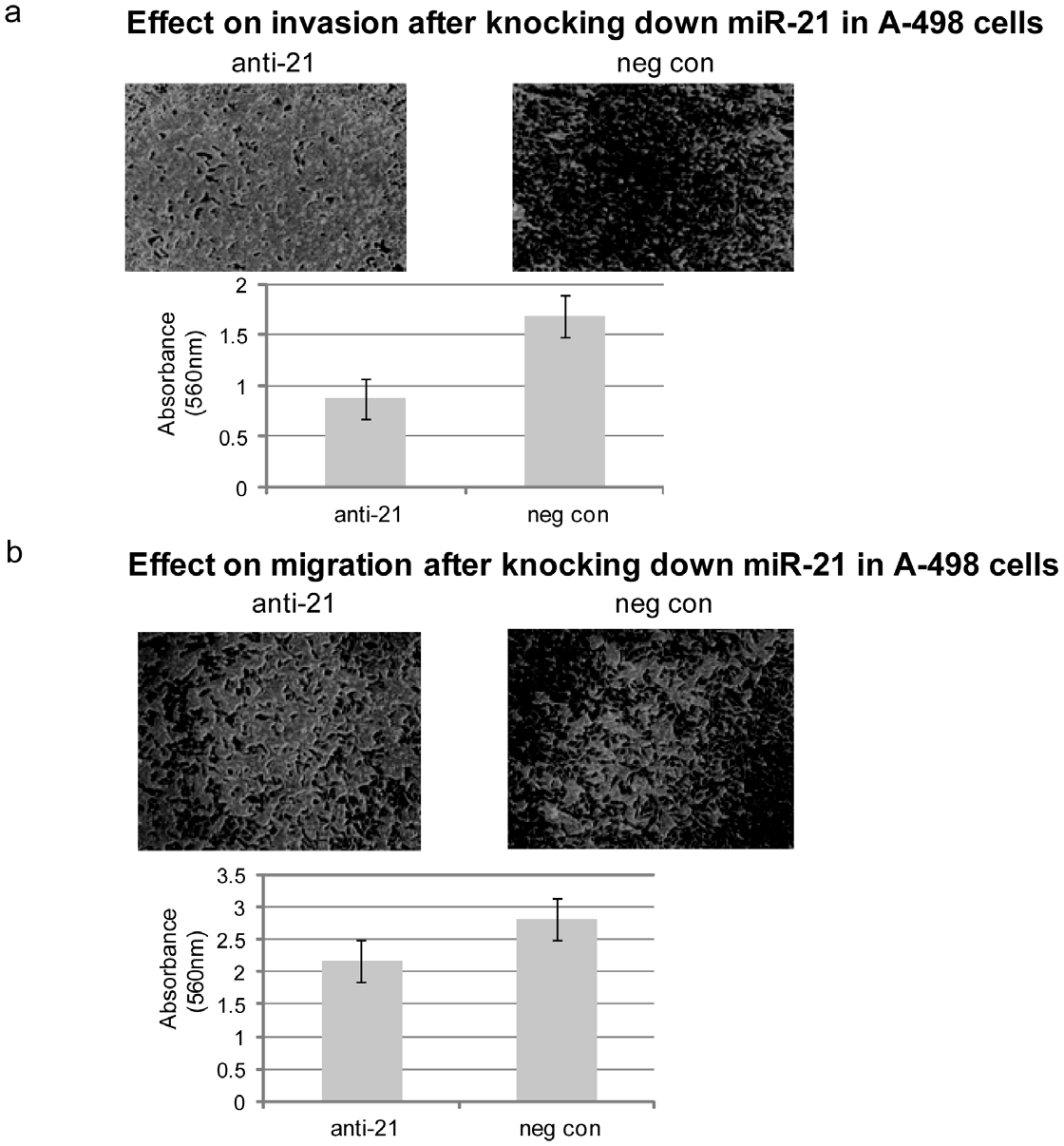
Effect on cell invasion and migration on A-498 cells after miR-21 inhibition. (a) Effect on cell invasion: Invasive properties of A-498 cells were decreased after the inhibition of miR-21 as compared to the negative control, Stained A-498 cells (Magnification-40×); Absorbance at 560 nm (p value 0.026). (b) Effect on cell migration: Migratory properties of A-498 cells were also decreased after the inhibition of miR-21 as compared to the negative control, Stained A-498 cells (Magnification-40×); Absorbance at 560 nm (p value 0.016).

To determine whether miR-21 affects renal cancer cell migration and invasion a cytoselect 24-well cell migration and invasion kit was used. A-498 cells were transfected with anti-miR-21 inhibitor and anti-miR-Negative control. Both A-498 cell invasion and migration were decreased as compared to negative control. Less absorbance was observed at 560 nm with anti-miR-21 transfected cells than the negative control in both the invasion and migration assays (Figures 3a and 3b). Inhibition of miR-21 expression had a more pronounced effect on invasion as compared to migration, (reduction of 50% vs 25%). Similar effects were observed in the case of Caki-2 cells in both invasion and migration assays (data not shown).

#### miR-21 increases the expression of cyclin dependent kinase inhibitor CDKN1A (p21)

**Figure 4:**
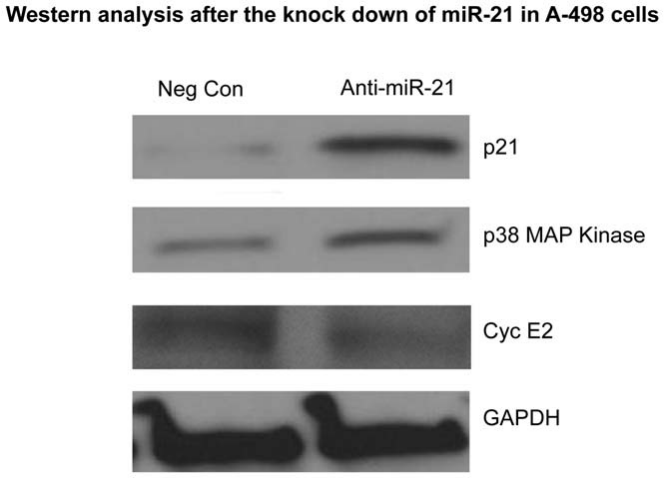
Western analysis after the knock down of miR-21 in A-498 cells. Expression of p21, p38MAP kinase and cyclin E2 after the inhibition of miR-21 in A-498 cells, as compared to negative control. GAPDH was used as a normalizing control. p21 and p38 MAP kinase were up regulated, whereas cyclin E2 expression was found to be down regulated.

To see the effect of miR-21 expression on different genes involved in the cell cycle and apoptosis we used PCR arrays. Since we saw a significant effect on the cell cycle by inhibition of miR-21 in A-498 cells, we first decided to use PCR arrays related to cell cycle genes. Cell cycle based PCR arrays showed changes in the expression of a number of genes. One of the genes up regulated in the PCR array after the inhibition of miR-21 was the cyclin dependent kinase inhibitor, CDKN1A also known as Cip1 or p21. Other PCR arrays showed a change in the expression of the gene p38 MAP kinase. Subsequently, we verified this result using MAP kinase based PCR arrays and obtained the same result (data not shown). Both results were validated by Western blotting (Figure 4). Western blotting experiments clearly show up regulation of both p21 and p38 MAP kinase genes with miR-21 inhibition as compared to the negative control. We also checked the expression of other genes related to the cell cycle and apoptosis and found downregulation of cyclin E2. We also looked at mRNA expression of cyclin dependent kinases, CDK2, CDK4, CDKE1 and CDKE2 and found their expression to be significantly reduced after inhibition of miR-21 (data not shown). In Western blot experiments we checked CDK4, E1 and E2 as CDK2 is already known to be affected by p21 gene [13], [14]. We observed a change in protein expression for only CDKE2.

### *In vitro* and *In vivo* effects of genistein on renal cancer cells

#### Genistein reduces the expression of miR-21 in mouse tumors

**Figure 5:**
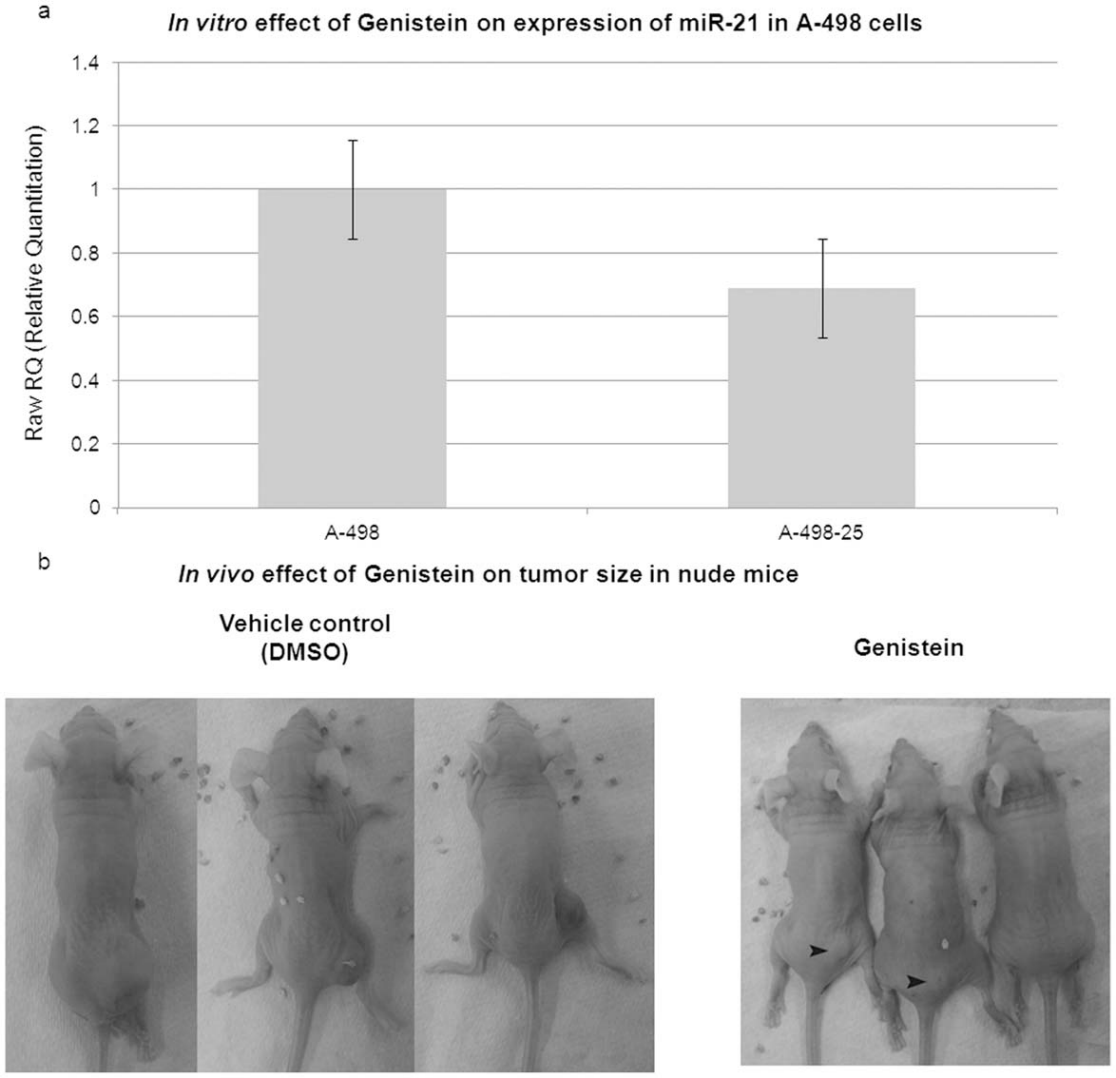
*In vitro* and *In vivo* effect of genistein on the expression of miR-21. (a) Effect of genistein (25 µM) on the expression of miR-21 in A-498 cells: miR-21 expression was found to be reduced by ∼30% in genistein treated A-498 cells as compared to untreated cells. (b) *In vivo* effect of genistein on tumor size in nude mice: Genistein was able to reduce tumor formation significantly (black arrows, right panel).

As mentioned earlier we checked the effect of a chemoprevention agent, genistein (a soy product), on the expression of miR-21 in nude mouse tumors. Firstly A-498 renal cancer cells were treated with 25 µM genistein for 4 days and subsequent to RNA extraction miR-21 expression was checked. The expression was observed to be reduced by 30% in genistein treated cells as compared to untreated ones (Figure 5a). To see the effect of genistein treatment on mice tumors and miR-21 expression A-498 cells were again treated with genistein (25 µM) and after 96 hours of treatment were injected subcutaneously into nude mice. Two sets of three mice each were used. In the DMSO control tumors the three mice developed significantly large sized tumors, approximately 800 mm^3^ in volume (Figure 5b, left panel), whereas with the genistein treated cells a small size tumor was observed in one out of three mice, approximately 25 mm^3^ in volume (Figure 5b right panel,). In the other two mice one of them had a very small tumor at the site of the injection (Figure 5b right panel) while the third mouse had no tumor. After the formation of palpable tumors with control cells, we excised the tumors and measured the expression of miR-21 subsequent to the tumor homogenization with Qiazol and RNA extraction. The expression of miR-21 was found to be reduced by ∼40% in genistein treated samples as compared to untreated ones (data not shown).

## Discussion

In the present study we provide evidence that up regulation of miR-21 in renal cell carcinoma is related to lower survival of kidney cancer patients. The expression of miR-21 in both kidney cancer cell lines and human kidney cancer tissue samples was found to be higher as compared to normal, which is in accordance with previous studies on renal cell carcinoma and also other types of cancer [15]. Our study shows for the first time that patients with a higher expression of miR-21 in renal cancer have a lower probability of survival. Also, lower expression of miR-21 leads to a higher survival rate. We also correlated the expression of miR-21 with the stage of the cancer for those patients that we had complete information regarding the stage (34/39). Our analysis showed that very high expression of miR-21 in patient samples led to an increase in stage of the disease. To elucidate the importance of miR-21 in renal cell carcinoma functional assays were performed. Cell viability assays showed an anti proliferative effect on A-498 cells after the inhibition of miR-21. The most significant change was observed in the cell cycle where the G0/G1 phase was increased dramatically (9.1%) with a concomitant reduction in the G2/M phase (6.8%). This led us to search for potential cell cycle genes affected by inhibition of miR-21 in A-498 cells. Cell cycle PCR array experiments showed that cDKN1A (p21) was up regulated after inhibition of miR-21 in A-498 cells. Previous studies using microarrays suggest that p21 expression positively correlates with the suppression of genes that are important for cell cycle progression [16]. p21 responds to a variety of stimuli that promotes growth-inhibitory activities depending on its ability to inhibit the activity of cyclin-dependent kinase, CDK2. Numerous studies have reported that the p21 gene has additional roles in cell cycle regulation which are independent of CDK2. Some examples are its association with the transcription factor E2F1 and the suppression of its transcriptional activity [17]. Furthermore, p21 also suppresses transcription factors STAT3 [18] and MYC [19]. In addition p21 also promotes cell cycle inhibition through the mediation of p53-dependent repression of genes such as CDC25C, CDC2, CCNB1 and BIRC5 also known as survivin [20], [21], [22]. Although p21 suppresses genes involved in cell cycle progression it also inhibits apoptosis [23]. When cells face genotoxic insults or microtubule-destabilizing agents, p21 protects them from apoptosis as an active cell cycle is required to sense these agents and trigger apoptosis. This may account for the not so dramatic increase in the number of cells undergoing apoptosis after the inhibition of miR-21 in A-498 cells as compared to changes in cell cycle progression Figure 2b. However, the effect of p21 is counteracted by several mechanisms which involves switching from cell cycle arrest to apoptosis by the selective transcriptional repression of p21, the selective activation of pro-apoptotic genes or defects in p21 expression downstream of p53 [24], [25], [26]. In certain cases p21 also promotes apoptosis through both p53-dependent and independent mechanisms under cellular stress but the mechanism of action is still unknown [27]. Thus we tried to see if p21 was a direct target of miR-21 using the 3′ luciferase assay but we could not find a direct interaction. Thus we checked the protein expression of different cyclin dependent kinases CDK4, E1 and E2 and could find a difference in only cyclinE2 (Figure 4).

Genistein, one of the principal soy isoflavones, has a wide array of chemopreventive actions. The anticancer effects of genistein have been ascribed to several signalling pathways and mechanisms that affect cell cycle arrest, apoptosis, invasion, metastasis and angiogenesis, attributes that could potentially prevent tumor initiation and progression [28], [29]. Genistein (40,5,7- trihydroxyflavone) is abundant in soy products and has been identified as an inhibitor of protein tyrosine kinases and thus may play a key role in cell growth and apoptosis [30], [31]. It has been reported to have estrogenic properties and anti-neoplastic activity in multiple tumor types [32]. It has also been found to have epigenetic effects in the mouse prostate [33]. In a previous study Hillman *et. al.* have shown that the combination of genistein with primary tumor irradiation acts more effectively as a therapeutic approach, in established kidney tumors, due to tumor growth being inhibited in both primary and metastatic sites. In their study genistein alone demonstrated a tendency to stimulate growth of primary kidney tumor and increase metastasis [34]. In our study we take a simpler approach and have shown that A-498 cells treated with genistein only formed reduced tumors as compared to vehicle controls.

miRNAs regulate gene expression by targeting genes containing complementary mRNA sequences [35]. Many cellular pathways are affected by the regulatory function of miRNAs and the most prominent of these pathways control developmental and oncogenic processes [36]. miR-21 was one of the first miRNAs detected in the human genome [37] and has been found to be overexpressed in a variety of cancer types [38]. There are a few studies documenting the functional relevance of miR-21 by showing the cause and effect relationship between miR-21 and neoplastic transformation. These studies have been done on glioblastoma [39], breast cancer [40], hepatocellular carcinoma [41], colorectal cancer [42] and in myeloma cells [43]. A recent report on the role of miR-21 in renal cancer shows that it modulates apoptosis through targeting several genes by Zhang *et.al* [44]. However to date there is no data which relates clinical correlation of miR-21 expression with the stage and survival of renal cancer patients. Moreover, we show the chemo preventive role of genistein in inhibiting tumor formation *in vivo* by decreasing miR-21 expression in mouse tumors. In conclusion this is the first report to correlate miR-21 levels with survival and stage of renal cancer patients and documents the oncogenic role of miR-21 through various cellular pathways.

## Materials and Methods

### Ethics Statement

Formalin-fixed, paraffin-embedded (FFPE) renal cancer samples were obtained from the San Francisco Veterans Affairs (VA) Medical Center. Written informed consent was obtained from all patients and the study was approved by the UCSF Committee on Human Research (Approval number: H9058-35751-01). All animal care was in accordance with the guidelines of the San Francisco Veterans Affairs Medical Center and the study was approved by the San Francisco VA IACUC (Protocol number: 08-003-01).

### Cell lines and cell culture

Human renal cancer cell lines A-498, Caki-1, Caki-2, and normal renal cell line HK-2 were obtained from the American Type Culture Collection (ATCC, Manassas, VA, USA). The cell lines were checked by ATCC through DNA (STR) profiling. Normal renal HK-2 cells were cultured as a monolayer in Keratinocyte Serum Free Medium (K-SFM) supplemented with 0.05 mg/ml bovine pituitary extract (BPE), 5 ng/ml human recombinant epidermal growth factor (EGF) (Life Technologies/Invitrogen, Carlsbad, CA, USA) and 10% fetal bovine serum (Atlanta Biologicals, Lawrenceville, GA, USA), 50 mg/ml penicillin and 50 mg/ml streptomycin (Invitrogen, Carlsbad, CA, USA). The renal cancer cell line A-498 was cultured as a monolayer in Eagle's Minimum Essential Medium, (UCSF Cell Culture Facility, San Francisco, CA, USA). Caki-1 and Caki-2 were cultured the same way in McCoy's5A media (UCSF Cell Culture Facility, San Francisco, CA, USA). All cell lines were maintained in an incubator with a humidified atmosphere of 95% air and 5% CO2 at 37°C. Subconfluent A-498cells (60–70% confluent) were treated with Genistein (25 µM). Genistein (Sigma-Aldrich Corp., St Louis, MO, USA) was dissolved in DMSO, and cells treated only with vehicle (DMSO) served as control. Fresh genistein was administered everyday along with a change of medium, and the cells incubated for 4 days.

### Quantitative real-time PCR

First-strand cDNA was prepared from total RNA (1 µg) using a reverse transcription system (Promega, Madison, WI, USA). Total RNA was extracted using an RNeasy mini kit from Qiagen (Valencia, CA, USA). For real-time polymerase chain reaction (PCR), complementary DNA was amplified with Inventoried Gene Assay Products containing two gene-specific primers and one TaqMan MGB probe (6-FAM dye-labeled) using a TaqMan Universal Fast PCR Master Mix in a 7500 Fast Real-Time PCR System (Applied Biosystems, Foster City, CA, USA). Thermal cycling conditions included 95°C for 20 seconds(s), 40 cycles of 95°C for 3 s and 60°C for 30 s according to the TaqMan Fast Universal PCR protocol. GAPDH was used as an endogenous control. For miRNA real-time experiments the cDNA strand was synthesised using Applied Biosystems Taqman MicroRNA Reverse Transcription kit, with 200 ng of total extracted miRNA. RNU48 was used as an endogenous control. For real-time PCR experiments, where miRNAs were isolated from formalin-fixed paraffin-embedded (FFPE) renal samples, let7-f was used as an endogenous control. Total miRNA was extracted from FFPE samples using a miRNA FFPE kit from Qiagen.

### PCR arrays through real-time PCR

To study the expression profile of genes involved in various cellular pathways, pathway-specific PCR arrays (cell cycle array, apoptosis array, MAP kinase array, SABioscience) were used according to the manufacturer's instructions. Data was analyzed with the manufacturer's software.

### Micro dissection of renal cancer tissues and RNA extraction from FFPE human renal tumor samples

Adjacent normal and cancerous renal tissues were obtained from 39 representative FFPE tissue blocks of radical nephrectomy specimens from the Pathology Department of the Veterans Affairs (VA) Medical Center of San Francisco. The blocks were from kidney cancer patients who were operated on at the VA Medical Center between 1980–2009. Sections (4 µm) of the blocks were prepared, H&E stained, and slides were reviewed by a board certified pathologist to mark the normal and cancer areas. Subsequently, 12 µm slides were made from the blocks and microdissection was performed using the marked H&E stained slides as a template. MicroRNA extraction was done using a Qiagen FFPE miRNA extraction kit. The levels of miR-21 were assessed by the Taqman miR assay as described above. Following PCR, relative miR-21 expression levels in cancerous regions were normalised to their adjacent normal counterparts.

### Cell transfection

A498 and Caki-2 cells were transiently transfected with either an inhibitor of miRNA-21 or anti-miR negative control #1 (from Ambion, Austin, TX, USA), using X-tremeGENE siRNA transfection reagent (Roche Diagnostics Indianapolis, IN, USA) according to the manufacturer's protocol. In brief, cells were seeded in 6 well plates (Nunc, Roskilde, Denmark) 24 h before transfection and were transiently transfected at a confluency of 40–50%. Mock transfection, with the transfection reagent, was also used as a control. The transfection mixture was dissolved in Opti-MEM serum-free media (UCSF Cell Culture Facility, San Francisco, CA, USA) and at the time of transfection cells were seeded in Eagle's Minimum Essential Medium (UCSF Cell culture facility), with 10% FBS (Atlanta Biologicals, Lawrenceville, GA, USA) and no antibiotics. On the following day the media was changed to Eagle's Minimum Essential Medium containing both FBS and 1% antibiotic (penicillin–streptomycin, 100×, UCSF Cell Culture Facility). Cells were pelleted after 72 h of transfection for flow cytometry, RNA and protein extraction.

### Cell cycle analysis

Cell cycle analysis was performed 72 h after transfection. The cells were harvested, washed with cold PBS, UCSF Cell Culture Facility, and resuspended in the nuclear stain 4′,6-diamidino-2-phenylindole (Beckman Coulter, Brea, CA, USA). Stained cells were immediately analysed with a flow cytometer (Cell Lab Quanta SC; Beckman Coulter).

### Apoptosis assay

For apoptosis, cells at 72 h after transfection were dual stained with the viability dye 7-amino-actinomycin D and Annexin V-FITC using an Annexin V-FITC/7-amino-actinomycin D kit (Beckman Coulter) according to the manufacturer's protocol. Stained cells were immediately analysed by flow cytometry (Cell Lab Quanta SC; Beckman Coulter).

### Migration and invasion assays

A cytoselect 24-well cell migration and invasion assay kit (Cell Biolabs, Inc., San Diego, CA) was used for migration and invasion assays at 72 hrs after transfection (8 µm, Colorimetric format) according to the manufacturer's protocol.

### Western analysis

Whole-cell extracts were prepared in radioimmunoprecipitation assay buffer (RIPA; Thermo Scientific, Rockford, IL, USA; 50 mmol l–1 Tris (pH 8.0), 150 mmol l–1 NaCl, 0.5% deoxycholate, 0.1% SDS and 1.0% NP-40) containing a protease inhibitor cocktail (Roche, Basel, Switzerland). Protein assays were performed using a BCA Protein assay kit (Pierce/Thermo Scientific, Rockford, IL, USA) according to the manufacturer's instructions. Total protein (40 µg) was electrophoresed in 12% SDS–PAGE gels, and Western blotting was carried out using standard protocols and detected by chemiluminescence. All antibodies, which included p21(cat. no. 2552), p38 MAPkinase (cat. no. 9212) and cyclinE2 (cat. no. 4132) were purchased from Cell Signaling (Danvers, MA, USA), whereas GAPDH (cat. no. sc-32233) was from Santa Cruz Biotechnology (Santa Cruz, CA).

### Genistein treatment of A-498 cells and sub-cutaneous injection in nude mice

As mentioned above, A-498 cells were treated with 25 µM genistein for 96 hours. DMSO (vehicle) treated cells served as the control. After 96 hours the cells were pelleted and ∼2 million cells were injected subcutaneously (100 µl) in the right flank of nude mice (4- to 5-week old; Charles River Laboratories). Two sets of mice, with three mice each, were used for this experiment. One set represented the vehicle (DMSO) controls and the other genistein treated cells. Tumors from control and genistein treated cells were excised after 8 weeks when the DMSO treated control mice developed tumors approximately 800 mm^3^ in volume (length-16 mm, breadth-10 mm). Total RNA was extracted by homogenizing the tumors in Qiazol and extracting RNA using the manufacturer's protocol (Qiagen). Expression of miR-21 was measured by real-time PCR.

### Statistical analysis

Statistical analysis was performed using StatView version 5.0 for Windows. Student's t-test was used to compare the different groups. p-values of <0.05 were regarded as statistically significant.

## Supporting Information

Figure S1 miR-21 levels in patients having low expression (<1.2): Low levels of miR-21 in patients with 100% survival.
(TIF)

Figure S2 miR-21 levels in patients having high expression (>1.2): High levels of miR-21 in patients with 50% survival.
(TIF)

Figure S3 Expression levels of miR-21 after knockdown in A-498 cells: The miR-21 level was reduced by more than 99% (1.0 to 0.030), as compared to the negative control.
(TIF)

Figure S4 Expression levels of miR-21 after knockdown in Caki-2 cells: The miR-21 level was reduced by more than 99% (1.0 to 0.011), as compared to the negative control.
(TIF)
