# Correction: Up-Regulation of MicroRNA-21 Correlates with Lower Kidney Cancer Survival

**DOI:** 10.1371/annotation/6662579f-3a41-4bce-9298-9d15f6582ef5

**Published:** 2012-04-09

**Authors:** Mohd Saif Zaman, Varahram Shahryari, Guoren Deng, Sobha Thamminana, Sharanjot Saini, Shahana Majid, Inik Chang, Hiroshi Hirata, Koji Ueno, Soichiro Yamamura, Kamaldeep Singh, Yuichiro Tanaka, Z. Laura Tabatabai, Rajvir Dahiya

There was a spelling error in the fifth author's name. The correct spelling is Sharanjot Saini.

