# Supplementary figures and images for: Up-Regulation of MicroRNA-21 Correlates with Lower Kidney Cancer Survival
Source: PLoS One. 2012 Feb 8;7(2):e31060. doi: 10.1371/journal.pone.0031060 (PMC3275568; doi:10.1371/journal.pone.0031060)

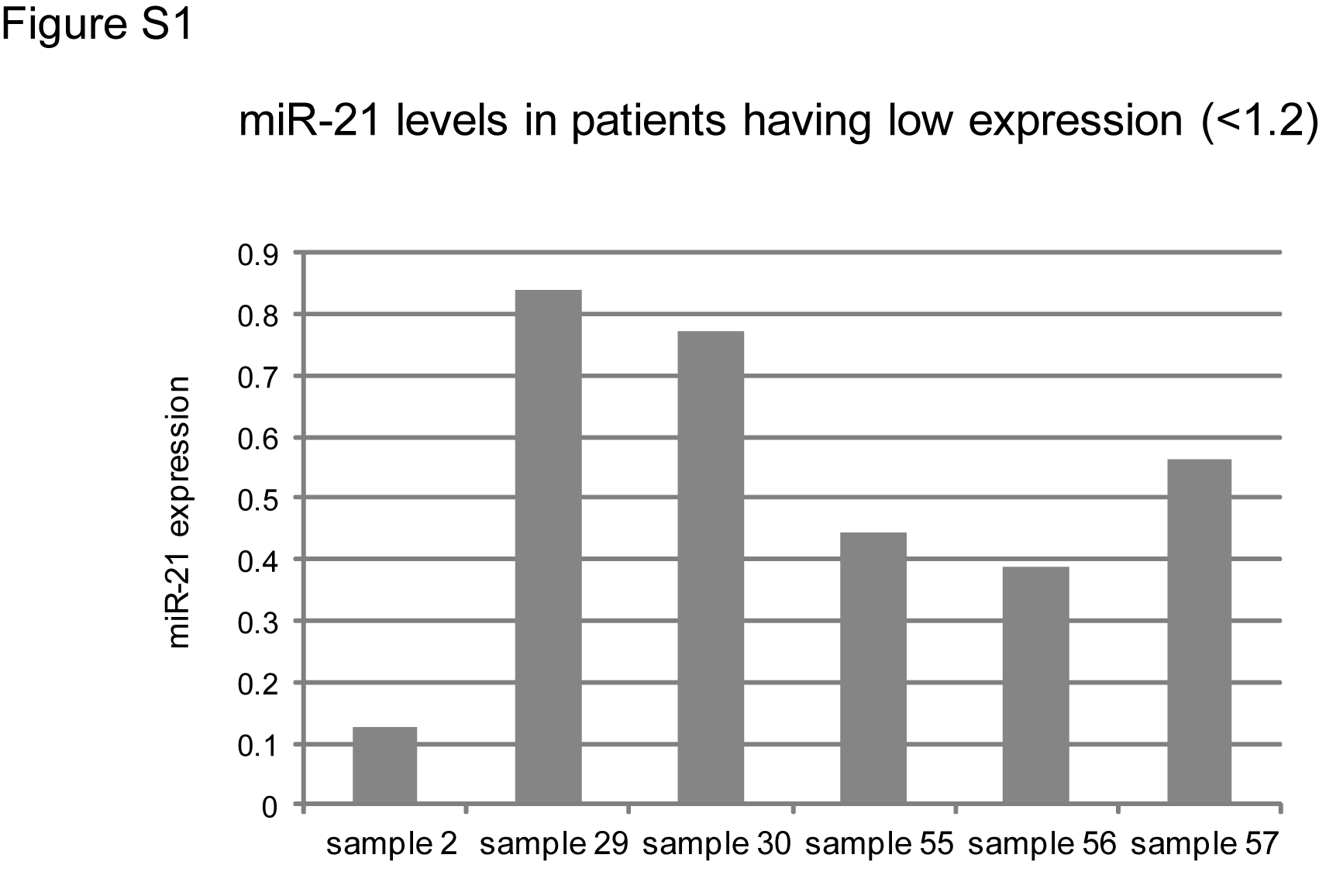

Supplement: Figure S1 — miR-21 levels in patients having low expression (<1.2): Low levels of miR-21 in patients with 100% survival. (TIF) [file pone.0031060.s001.tif]

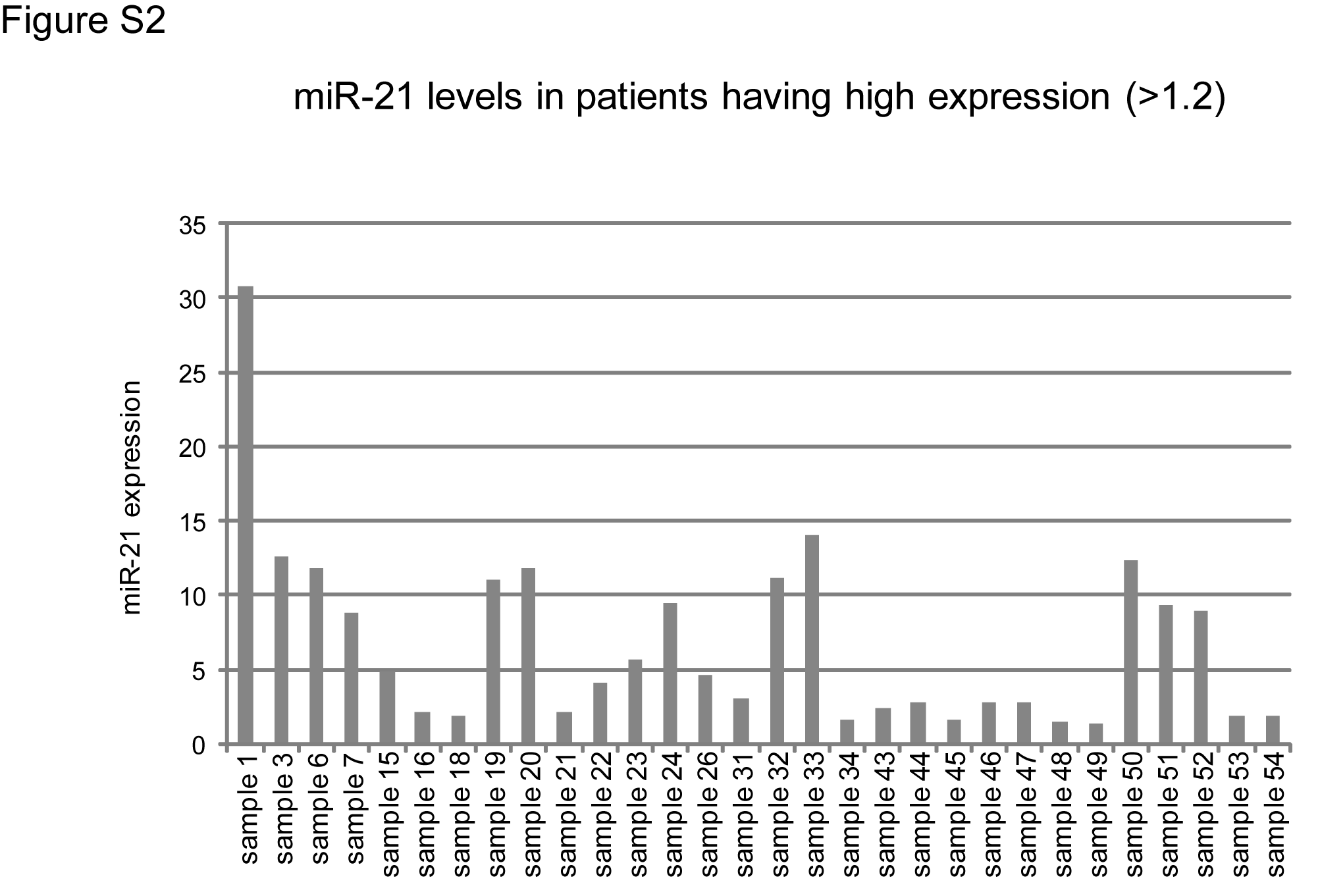

Supplement: Figure S2 — miR-21 levels in patients having high expression (>1.2): High levels of miR-21 in patients with 50% survival. (TIF) [file pone.0031060.s002.tif]

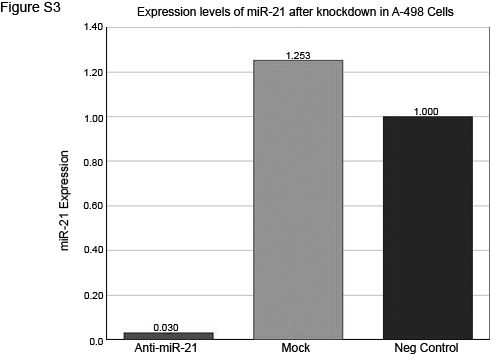

Supplement: Figure S3 — Expression levels of miR-21 after knockdown in A-498 cells: The miR-21 level was reduced by more than 99% (1.0 to 0.030), as compared to the negative control. (TIF) [file pone.0031060.s003.tif]

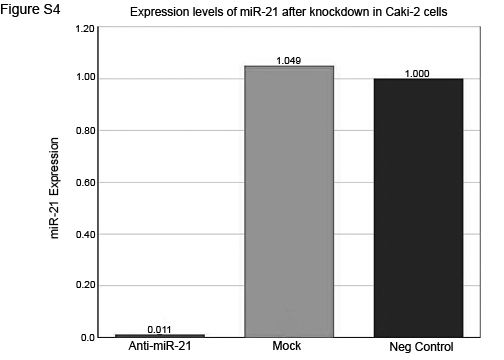

Supplement: Figure S4 — Expression levels of miR-21 after knockdown in Caki-2 cells: The miR-21 level was reduced by more than 99% (1.0 to 0.011), as compared to the negative control. (TIF) [file pone.0031060.s004.tif]
